# 

**Published:** 1873-05

**Authors:** 


### TABLE OF CONTENTS.

### ORIGINAL COMMUNICATIONS.

Normal Ovariotomy. By Robert Battey, M.D., Rome, Ga. (Read Before the Georgia Medical Association, Atlanta, Ga., April, 1873)............ 65
Cerebro-Spinal Meningitis—Its Pathology and Treatment. By J. G. Westmoreland, M.D.................................................. 84
Hints to Young Practitioners—No. VI. By Senex..................... 91
Abstracts from German Journals. By Ch. Rauschenberg, M. D......... 92
(On the Catarrh of the Genital Organs of the Female—Fluor Albus.)

### FOREIGN CORRESPONDENCE.

Opaque Optic Nerve Fibres in the Retina. By A. W. Calhoun, M. D.....105

### EDITORIAL AND MISCELLANEOUS.

Medical Organization..............................................113
American Medical Association......................................115
Medical Association of Georgia....................................117
International Medical Congress....................................118
The First Tracheotomy with the Aid of the Galvano-Cautery, etc.......119
Aloes in Hemorrhoids..............................................121
Nitras Argenti to the Uterine Cavity..............................121
Removal of a Needle from the Heart................................122
Ventilation and Warming...........................................122

ROBERT BATTEY, M.D., Rome, Ga.

ASSOCIATE EDITORS :

CH. RAUSCHENBERG, M.D.,

